# Crystal structures of two isomers of 1-(naphthalen-1-yl)ethanol

**DOI:** 10.1107/S2056989025009533

**Published:** 2025-11-11

**Authors:** Christopher Golz

**Affiliations:** a Georg-August-Universität Göttingen, Institut für Organische und Biomolekulare Chemie, Tammannstrasse 2, D-37077 Göttingen, Germany; University of Durham, United Kingdom

**Keywords:** crystal structure, hydrogen bonding, π–π-inter­actions, Hirshfeld surfaces, energy lattice

## Abstract

Crystal structures, Hirshfeld surfaces and energy lattices of (*S*)-1-(naphthalen-1-yl)ethanol (**1**) and (*R*)-1-(naphthalen-2-yl)ethanol (**2**), both C_12_H_12_O, were studied to understand much the lower crystallization propensity of the latter. In both structures, mol­ecules are linked by strong hydrogen bonds into helical chains where Coulombic inter­actions expectedly dominate, but dispersive inter­actions of **1** and **2** differ significantly, resulting in large gaps in the total energy lattice of **2**. The poor crystallization and and higher *Z*′ (4 *vs* 2 in structure **1**) of **2** can be explained by frustration between supra­molecular synthons (⋯O—H⋯O—H⋯ hydrogen-bonding chain *vs* π–π- inter­actions between naphthalene moieties). The study provides new insights into the supra­molecular inter­actions and crystal packing of regioisomeric naphthalenyl-ethanol compounds, which may have implications for the design of new materials with tailored properties.

## Abstract 2

Crystal structures, Hirshfeld surfaces and energy lattices of (*S*)-1-(naphthalen-1-yl)ethanol (**1**) and (*R*)-1-(naphthalen-2-yl)ethanol (**2**), both C_12_H_12_O, were studied to understand much lower crystallization propensity of the latter. The study provides new insights into the supra­molecular inter­actions and crystal packing of regioisomeric naphthalenyl-ethanol compounds, which may have implications for the design of new materials with tailored properties.

## 1. Chemical context

**1** and **2** are chiral aromatic alcohols, mutually regioisomeric and differing by the attachment position of the ethanol moiety to the naphthalene group. Such rather simple monoalcohols are inter­esting objects to explore certain packing principles and are often discussed in this connection; monoalcohols specifically appear to behave in a rather systematic way governed by (i) the propensity to form strongly directional hydrogen bonds that link OH groups in chains or rings and (ii) the sterical bulkiness of the organic residue (Brock & Duncan, 1994 ▸). This also leads to frequent occurrence of Z′>1 and extensive polymorphism (Steed & Steed, 2015 ▸; Taylor *et al.*, 2016 ▸). One common approach is to group certain inter­actions into supra­molecular synthons, which prefer definite relative orientations (Anderson *et al.*, 2008 ▸). It is further suggested that, in cases where the synthons are not acting synergistically, structural frustration is building up leading to the formation of high-*Z*′ structures. For the alcohols studied here, the synthons are the hydrogen bonding between hy­droxy groups and the π–π-inter­actions between naphthalene groups. To explain the contrasting crystallization behavior, with **1** forming very readily sizable single crystals while **2** only reluctantly crystallizing at all, the single crystal structures of both were determined and compared.
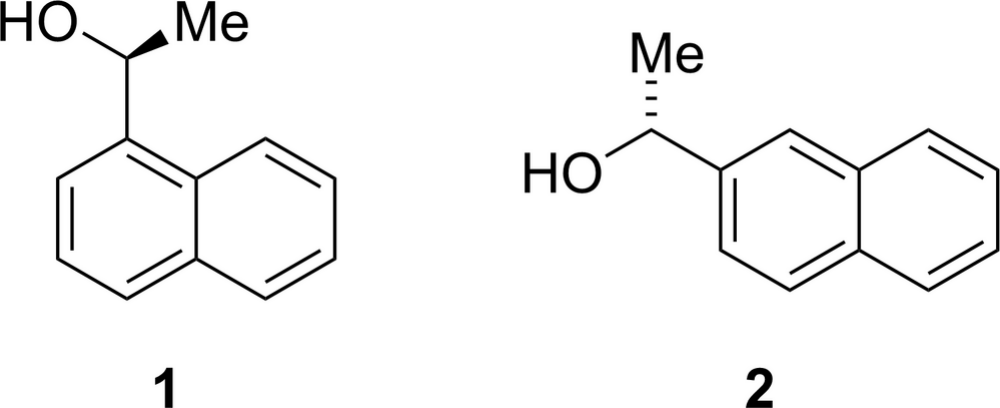


## 2. Structural commentary

**Figure 1:**
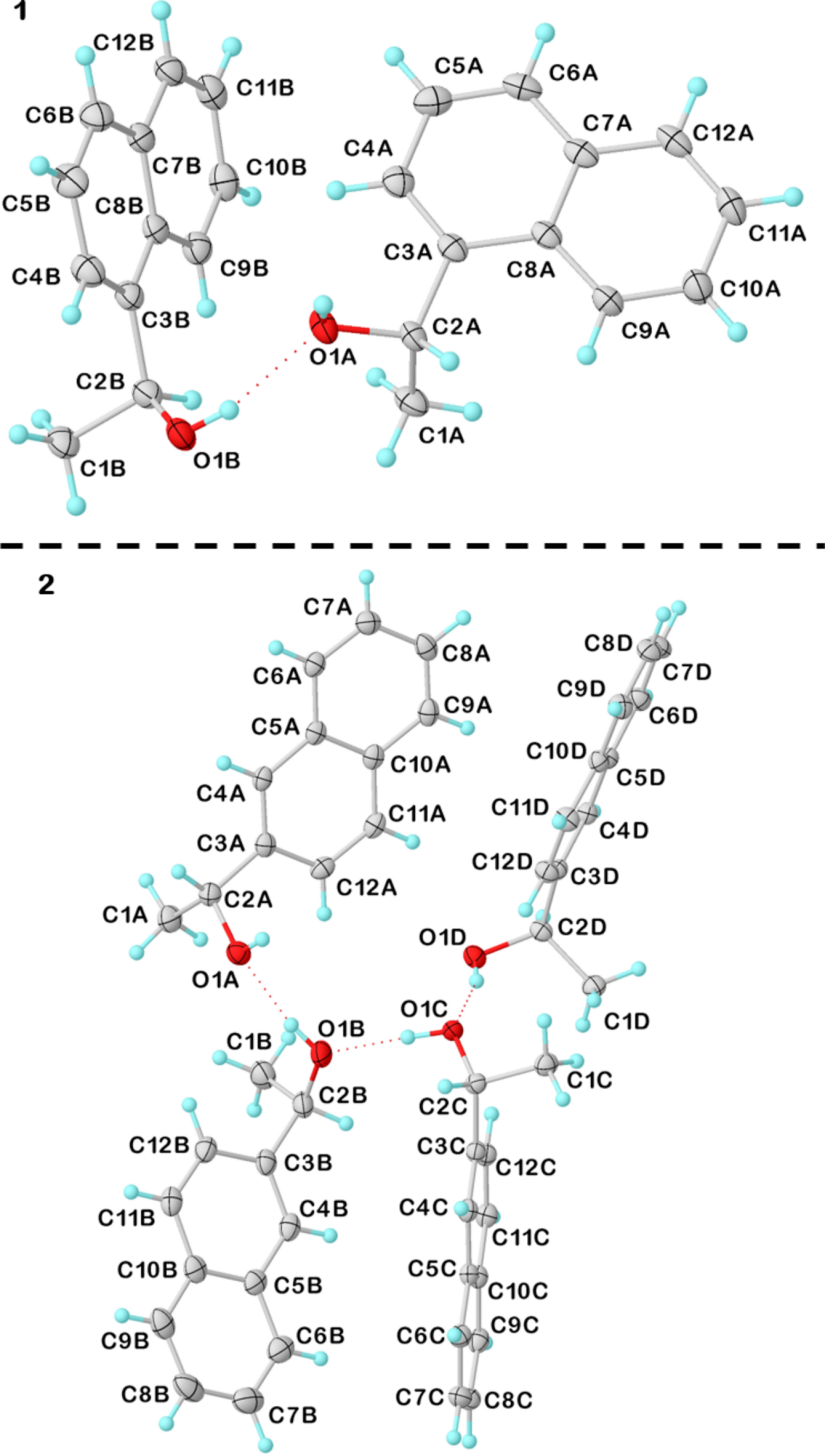
Mol­ecular structures and atom-numbering schemes of alcohols **1** (top) and **2** (bottom), showing a full asymmetric unit for either. Independent mol­ecules label carry the suffix A to D, respectively. Atomic displacement ellipsoids are drawn at the 50% probability level.

**Figure 2:**
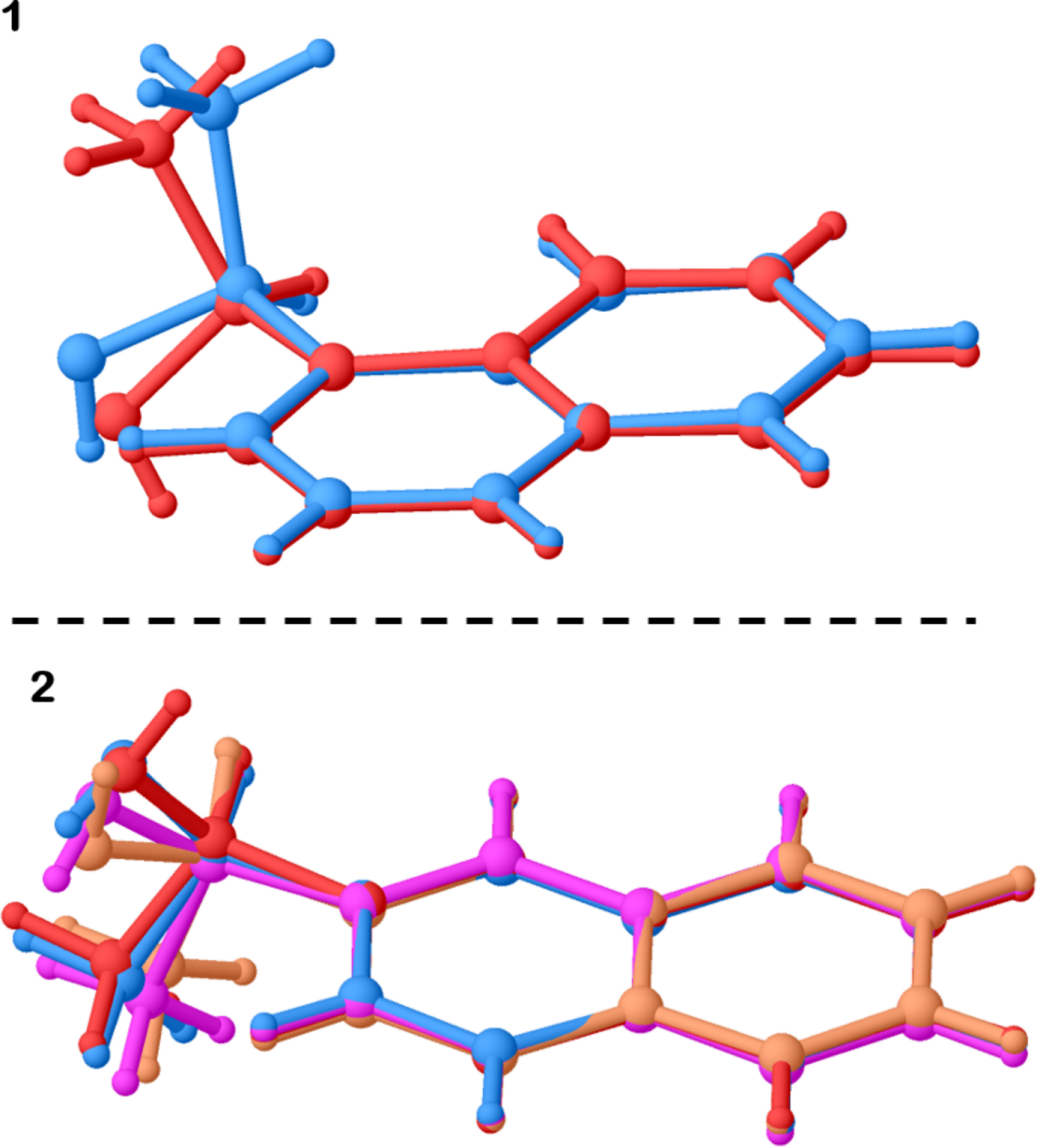
Overlay of the independent mol­ecules of **1** (top) and **2** (bottom) in which the naphthalene moieties were aligned. Mol­ecules *A* are shown in red, *B* in blue, *C* in orange and *D* in magenta.

Alcohol **1** crystallizes in the ortho­rhom­bic space group *P*2_1_2_1_2_1_ with two independent mol­ecules in the asymmetric unit, while **2** crystallizes in monoclinic *P*2_1_ with four independent mol­ecules (see Fig. 1 ▸). The independent mol­ecules differ mainly in the conformation of the ethanol group (see the mol­ecular overlay in Fig. 2 ▸). For **1** the difference is limited to a *ca*. 20° rotation around the C2—C3 bond. In structure **2**, mol­ecules *B* and *D* have their hy­droxy group O—H bond oriented roughly in the plane of the naphthalene moiety, while in mol­ecules *A* and *C* it is oriented almost perpendicularly to this plane, with consequences for the packing (see Section 3).

## 3. Supra­molecular features

**Figure 3:**
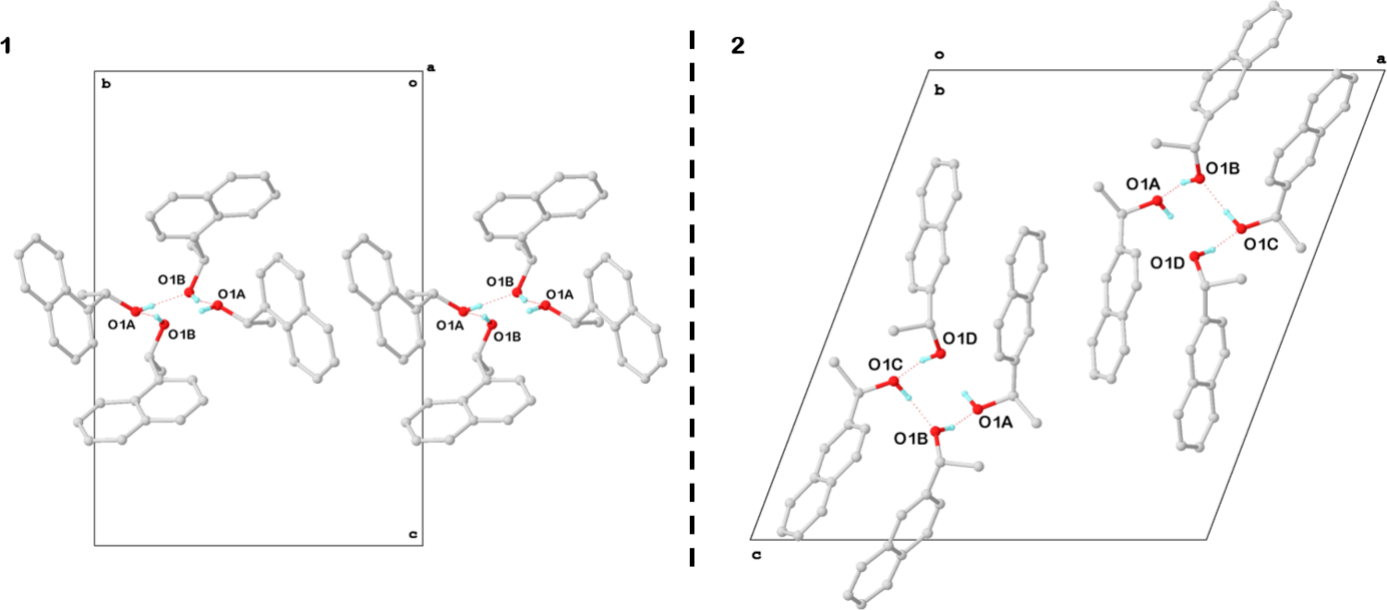
Packing diagrams for **1** and **2**, showing the hydrogen-bond chain motif.

At first glance, both alcohols form apparently similar columns of mol­ecules with a central hydrogen-bonding chain following a helical motif that can be described by a pseudo-4_1_ screw (see Fig. 3 ▸). Two differences become notable at closer inspection. Firstly, the naphthalene groups are arranged differently. In **1**, their orientations approximately follow the same pseudo-4_1_ motif, while in **2** they do not. Instead, the naphthalene and methyl groups each are arranged on opposite faces of the formed column. Secondly, the hydrogen bonds vary in length (defined as the donor-acceptor O⋯O distance) differently in both structures. In **1**, it is alternating between one short and one long hydrogen bond (Table 1 ▸) while in **2**, there are three longer hydrogen bonds, followed by one particularly short one of 2.665 (2) Å (Table 2 ▸).

### 3.1. Hirshfeld surface and energy lattice analysis

**Figure 4:**
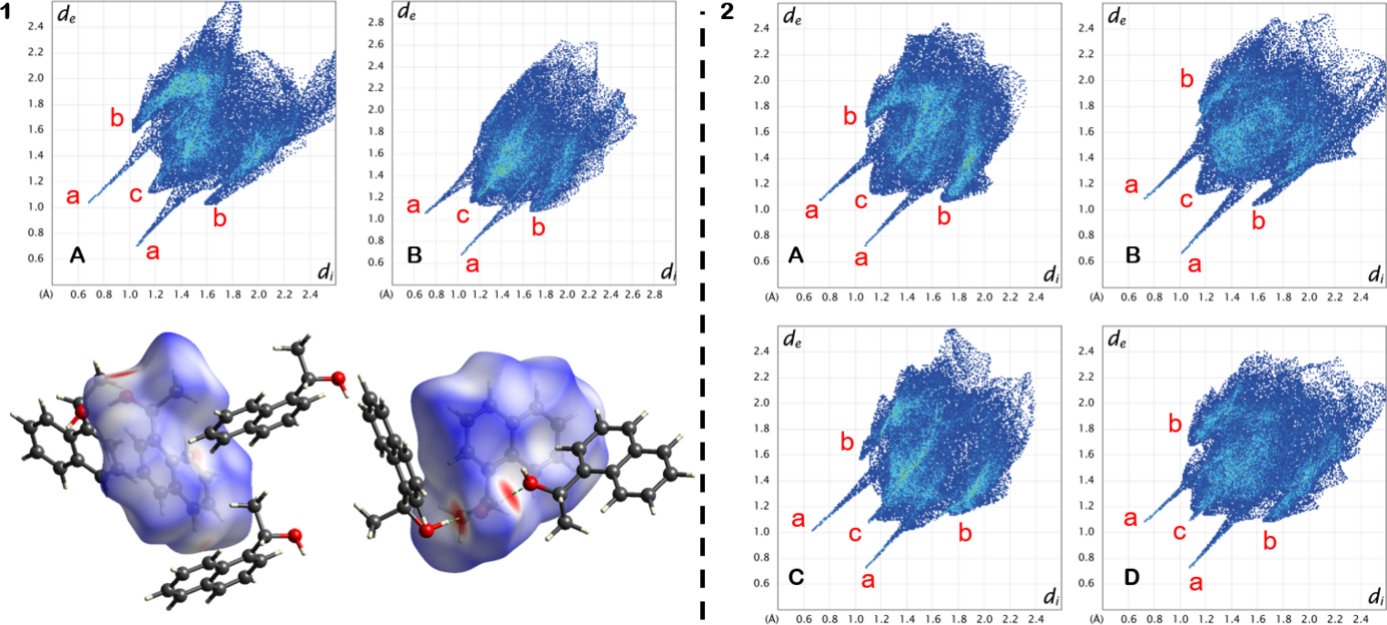
Hirshfeld surface and fingerprint plots for **1** and **2**. Red letters indicate close inter­actions H⋯O (*a*), H⋯C (*b*) and H⋯H (*c*).

To better understand how the mol­ecules inter­act within the columns, the corresponding Hirshfeld surfaces were computed with *CrystalExplorer21* and analyzed (Spackman *et al.*, 2021 ▸). The most prominent red spot on the Hirshfeld surface is clearly indicating the hydrogen bond between the adjacent hy­droxy groups, and the fingerprint plot shows the corresponding sharp spike (marked ‘a’ in Fig. 4 ▸). An inter­esting feature in **1** is the peripheral spike of H⋯C contacts (marked ‘b’ in Fig. 4 ▸), which indicates C—H⋯π inter­actions. This feature is not symmetrically present for both independent mol­ecules of **1**, indicating that some of the close C—H⋯π inter­actions occur between two *A* mol­ecules when they are symmetric contacts, and between *A* and *B* when they are not symmetric, whereas mol­ecule *B* acts mostly as an acceptor. Upon further inspection of these contacts, they appear as inter-column edge-to-face inter­actions (Martinez & Iverson, 2012 ▸).

**Figure 5:**
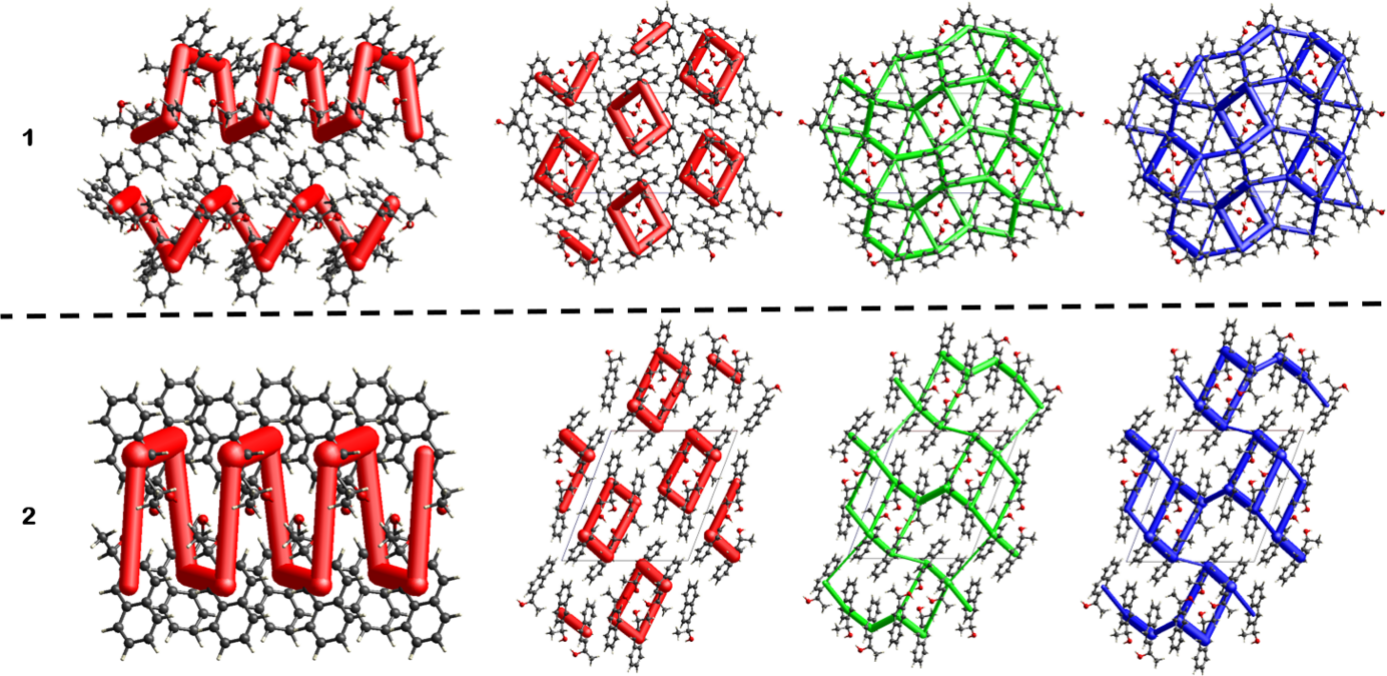
Calculated energy lattices (CE-B3LYP) for **1** and **2**, viewed perpendicular to (leftmost figures) and down the propagation axes of the pseudo 4_1_-screw. Coulombic inter­actions are represented by red tubes, dispersive inter­actions by green and total energy by blue ones. The tube scale is set to 150 and the cut-off for weak inter­actions is set to 10 kJ mol^−1^.

Further insight into the relative strength of these inter­actions can be obtained from comparison of the energy lattices (Mackenzie *et al.*, 2017 ▸), see Fig. 5 ▸. In both structures, the Coulombic inter­actions are clearly dominating along the hydrogen-bonding direction (Table 3 ▸), replicating the helical column structure discussed above. In terms of absolute energy, these are also the strongest inter­actions. A noticeable difference becomes visible when the dispersive inter­actions are scrutinized. Although weaker in absolute strength than the electrostatics, they are more numerous because they connect the columns. The dispersive intra-column inter­actions, following the hydrogen bonding, are on a similar scale as the inter-column ones. Inter­estingly, the inter­actions add up differently: noticeable gaps are present in the energy framework of **2**, which is not the case in **1**. This might indicate the aforementioned frustration between supra­molecular synthons in **2** (Anderson *et al.*, 2008 ▸) and serve to explain the higher *Z*′ and lower observed crystallization propensity.

## 4. Database survey

Several related arenyl methanols can be found in the Cambridge Crystallographic Database (CSD ver. 5.43; Groom *et al.*, 2016 ▸), all featuring the hydrogen-bond chain motif. In phenanthren-4-yl-methanol (FUGZAI; Gerkin, 2000 ▸), a very similar packing arrangement is found as in **1** and **2**, but in a more ideal realization with *Z*′ = 1 in space group *I4*_1_/*a*. Herein, the hydrogen bond column is following a perfect 4_1_ screw symmetry. On the other hand, the organization of the hydrogen bond column appears to be more distorted when the π-system is enlarged: in anthracen-9-yl-methanol (VAFMUK; Sweeting & Rheingold, 1988 ▸; Islor *et al.*, 2013 ▸) the packing features columns that are not following any screw but a glide operation. In pyren-1-yl-methanol (DUPBAS; Gruber *et al.*, 2010 ▸; Morales-Espinoza *et al.*, 2011 ▸) the mol­ecules appear to favor the formation of discrete π-stacked dimers, that further stretches the pseudo-4_1_ screw motif along the propagation direction. This is notable when comparing the period lengths, involving four mol­ecules per 360° rotation of the helix, *viz*. 6.03 Å in **2**, 7.75 Å in **1**, 8.30 Å in FUGZAI and 8.86 Å DUPBAS.

Furthermore, the racemic structure of **2** is known (TAZTAQ; Staples & George, 2005 ▸); it contains discrete centrosymmetric tetra­mers rather than helical columns. The mol­ecular volumes of racemic and enanti­opure structures of **2** are 230.6 Å^3^ (193 K) *versus* 233.6 Å^3^ (100 K), respectively. The former is more dense and thus in accordance with Wallach’s rule (Brock *et al.*, 1991 ▸).

## 5. Synthesis and crystallization

Compounds **1** and **2** were both purchased from BLD Pharmatech GmbH. **1** was received as crystalline material from which suitable single crystals could be taken without further recrystallization. **2** was received as semi-amorphous solid and its recrystallization from various organic solvents yielded finely fibrous material. Single crystals of **2** suitable for diffraction experiments were grown from a solution in ethanol/water over the course of one week.

## 6. Refinement

Crystal data, data collection and structure refinement details are summarized in Table 4 ▸. C-bound H atoms were placed geometrically and treated as riding atoms, with C—H = 0.95 Å (aromatic), 0.98 Å (meth­yl), and 1.00 Å (*tert*-C). *U*_iso_(H) was set to 1.5*U*_eq_(C) for methyl hydrogen atoms and 1.2*U*_eq_(C) otherwise. The positions of hydroxyl H atoms were refined freely, while *U*_iso_(H) were set to 1.5*U*_eq_(O).

## Supplementary Material

Crystal structure: contains datablock(s) 1, 2. DOI: 10.1107/S2056989025009533/zv2039sup1.cif

Structure factors: contains datablock(s) 1. DOI: 10.1107/S2056989025009533/zv20391sup2.hkl

Structure factors: contains datablock(s) 2. DOI: 10.1107/S2056989025009533/zv20392sup3.hkl

Supporting information file. DOI: 10.1107/S2056989025009533/zv20391sup4.cml

Supporting information file. DOI: 10.1107/S2056989025009533/zv20392sup5.cml

CCDC references: 2498864, 2498863

Additional supporting information:  crystallographic information; 3D view; checkCIF report

## Figures and Tables

**Table 1:** Hydrogen-bond geometry (Å, °) for **1**[Chem scheme1]

*D*—H⋯*A*	*D*—H	H⋯*A*	*D*⋯*A*	*D*—H⋯*A*
O1*A*—H1*A*⋯O1*B*^i^	0.85 (2)	1.84 (2)	2.6849 (15)	172 (2)
O1*B*—H1*B*⋯O1*A*	0.85 (2)	1.89 (2)	2.7119 (14)	165 (2)

Symmetry code: (i) 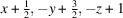.

**Table 2:** Hydrogen-bond geometry (Å, °) for **2**[Chem scheme1]

*D*—H⋯*A*	*D*—H	H⋯*A*	*D*⋯*A*	*D*—H⋯*A*
O1*A*—H1*A*⋯O1*D*^i^	0.86 (3)	1.94 (3)	2.7890 (18)	171 (2)
O1*B*—H1*B*⋯O1*A*	0.84 (3)	1.95 (3)	2.761 (2)	162 (3)
O1*C*—H1*C*⋯O1*B*	0.91 (3)	1.76 (3)	2.6648 (18)	177 (2)
O1*D*—H1*D*⋯O1*C*	0.87 (3)	1.93 (3)	2.7747 (18)	163 (2)

Symmetry code: (i) 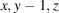.

**Table 3:** Calculated inter­action energies (kJ mol^−1^) between hydrogen-bonded mol­ecules

Comp	Path	*E*_ele_	*E*_pol_	*E*_dis_	*E*_rep_	*E*_tot_
1	*A*⋯*B*^i^	−50.8	−10.9	−24.8	72.3	−38.7
1	*B*⋯*A*	−45.3	−10.6	−33.9	69.9	−42.0
2	*A*⋯*D*^ii^	−47.3	−10.4	−41.2	69.5	−50.6
2	*B*⋯*A*	−43.1	−9.5	−19.8	55.4	−35.6
2	*C*⋯*B*	−61.0	−13.5	−37.7	88.2	−52.8
2	*D*⋯*C*	−40.6	−8.8	−21.2	54.5	−34.2

Symmetry codes: (i) *x* + 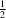, −*y* + 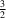, −*z* + 1, (ii) *x*, *y* − 1, *z*.

**Table 4:** Experimental details

	**1**	**2**
Crystal data		
Chemical formula	C_12_H_12_O	C_12_H_12_O
*M*_r_	172.22	172.22
Crystal system, space group	Orthorhombic, *P*2_1_2_1_2_1_	Monoclinic, *P*2_1_
Temperature (K)	100	100
*a*, *b*, *c* (Å)	7.7519 (7), 12.9750 (12), 18.794 (3)	17.3408 (5), 6.0327 (2), 19.1125 (5)
α, β, γ (°)	90, 90, 90	90, 110.821 (1), 90
*V* (Å^3^)	1890.3 (4)	1868.82 (10)
*Z*	8	8
Radiation type	Cu *K*α	Cu *K*α
μ (mm^−1^)	0.59	0.60
Crystal size (mm)	0.41 × 0.39 × 0.23	0.56 × 0.05 × 0.04

Data collection		
Diffractometer	Bruker D8 VENTURE dual wavelength Mo/Cu	Bruker D8 VENTURE dual wavelength Mo/Cu
Absorption correction	Numerical (*SADABS*; Krause *et al.*, 2015 ▸)	Numerical (*SADABS*; Krause *et al.*, 2015 ▸)
*T*_min_, *T*_max_	0.771, 0.982	0.355, 0.473
No. of measured, independent and observed [*I* > 2σ(*I*)] reflections	63841, 4063, 4058	70639, 7878, 7515
*R*_int_	0.040	0.051
(sin θ/λ)_max_ (Å^−1^)	0.637	0.637

Refinement		
*R*[*F*^2^ > 2σ(*F*^2^)], *wR*(*F*^2^), *S*	0.027, 0.068, 1.04	0.035, 0.096, 1.03
No. of reflections	4063	7878
No. of parameters	244	486
No. of restraints	0	1
H-atom treatment	H atoms treated by a mixture of independent and constrained refinement	H atoms treated by a mixture of independent and constrained refinement
Δρ_max_, Δρ_min_ (e Å^−3^)	0.19, −0.12	0.19, −0.16
Absolute structure	Flack *x* determined using 1715 quotients [(*I*^+^)−(*I*^−^)]/[(*I*^+^)+(*I*^−^)] (Parsons *et al.*, 2013 ▸)	Flack *x* determined using 3213 quotients [(*I*^+^)−(*I*^−^)]/[(*I*^+^)+(*I*^−^)] (Parsons *et al.*, 2013 ▸)
Absolute structure parameter	0.04 (4)	0.00 (15)

Computer programs: *APEX6* (Bruker, 2024 ▸), *SAINT* (Bruker, 2016 ▸), *SHELXT* (Sheldrick, 2015*a* ▸), *SHELXL* (Sheldrick, 2015*b* ▸) and *OLEX2* (Dolomanov *et al.*, 2009 ▸).

## supplementary crystallographic information

### (S)-1-(Naphthalen-1-yl)ethanol (1) Crystal data

**Table:**

C_12_H_12_O	*D*_x_ = 1.210 Mg m^−^^3^
*M**_r_* = 172.22	Cu *K*α radiation, λ = 1.54178 Å
Orthorhombic, *P*2_1_2_1_2_1_	Cell parameters from 9660 reflections
*a* = 7.7519 (7) Å	θ = 5.8–78.9°
*b* = 12.9750 (12) Å	µ = 0.59 mm^−^^1^
*c* = 18.794 (3) Å	*T* = 100 K
*V* = 1890.3 (4) Å^3^	Block, colourless
*Z* = 8	0.41 × 0.39 × 0.23 mm
*F*(000) = 736	

### (S)-1-(Naphthalen-1-yl)ethanol (1) Data collection

**Table:**

Bruker D8 VENTURE dual wavelength Mo/Cu diffractometer	4063 independent reflections
Radiation source: microfocus sealed X-ray tube, Incoatec Iµs	4058 reflections with *I* > 2σ(*I*)
Mirror optics monochromator	*R*_int_ = 0.040
Detector resolution: 7.41 pixels mm^-1^	θ_max_ = 79.1°, θ_min_ = 4.1°
ω and φ scans	*h* = −9→9
Absorption correction: numerical (SADABS; Krause *et al.*, 2015)	*k* = −16→16
*T*_min_ = 0.771, *T*_max_ = 0.982	*l* = −23→23
63841 measured reflections	

### (S)-1-(Naphthalen-1-yl)ethanol (1) Refinement

**Table:**

Refinement on *F*^2^	Hydrogen site location: mixed
Least-squares matrix: full	H atoms treated by a mixture of independent and constrained refinement
*R*[*F*^2^ > 2σ(*F*^2^)] = 0.027	*w* = 1/[σ^2^(*F*_o_^2^) + (0.0307*P*)^2^ + 0.3995*P*] where *P* = (*F*_o_^2^ + 2*F*_c_^2^)/3
*wR*(*F*^2^) = 0.068	(Δ/σ)_max_ < 0.001
*S* = 1.04	Δρ_max_ = 0.19 e Å^−^^3^
4063 reflections	Δρ_min_ = −0.12 e Å^−^^3^
244 parameters	Extinction correction: SHELXL-2018/3 (Sheldrick 2015b), Fc^*^=kFc[1+0.001xFc^2^λ^3^/sin(2θ)]^-1/4^
0 restraints	Extinction coefficient: 0.0034 (4)
Primary atom site location: structure-invariant direct methods	Absolute structure: Flack *x* determined using 1715 quotients [(*I*^+^)-(*I*^-^)]/[(*I*^+^)+(*I*^-^)] (Parsons *et al.*, 2013)
Secondary atom site location: difference Fourier map	Absolute structure parameter: 0.04 (4)

### (S)-1-(Naphthalen-1-yl)ethanol (1) Special details

**Table:**

Geometry. All esds (except the esd in the dihedral angle between two l.s. planes) are estimated using the full covariance matrix. The cell esds are taken into account individually in the estimation of esds in distances, angles and torsion angles; correlations between esds in cell parameters are only used when they are defined by crystal symmetry. An approximate (isotropic) treatment of cell esds is used for estimating esds involving l.s. planes.
Refinement. 1. Fixed Uiso At 1.2 times of: All C(H) groups At 1.5 times of: All C(H,H,H) groups, All O(H) groups 2.a Ternary CH refined with riding coordinates: C2A(H2A), C2B(H2B) 2.b Aromatic/amide H refined with riding coordinates: C4A(H4A), C5A(H5A), C6A(H6A), C9A(H9A), C10A(H10A), C11A(H11A), C12A(H12A), C4B(H4B), C5B(H5B), C6B(H6B), C9B(H9B), C10B(H10B), C11B(H11B), C12B(H12B) 2.c Idealised Me refined as rotating group: C1A(H1AA,H1AB,H1AC), C1B(H1BA,H1BB,H1BC)

### (S)-1-(Naphthalen-1-yl)ethanol (1) Fractional atomic coordinates and isotropic or equivalent isotropic displacement parameters (Å^2^)

**Table:**

	*x*	*y*	*z*	*U*_iso_*/*U*_eq_	
O1A	0.74231 (13)	0.62472 (8)	0.49349 (6)	0.0251 (2)	
H1A	0.811 (3)	0.6732 (16)	0.5051 (11)	0.038*	
C1A	0.63141 (19)	0.46242 (12)	0.52883 (9)	0.0269 (3)	
H1AA	0.532808	0.497591	0.550635	0.040*	
H1AB	0.656238	0.398943	0.555173	0.040*	
H1AC	0.604083	0.445431	0.479294	0.040*	
C2A	0.78844 (17)	0.53276 (10)	0.53101 (8)	0.0213 (3)	
H2A	0.815388	0.550398	0.581602	0.026*	
C3A	0.94438 (17)	0.48044 (10)	0.49746 (8)	0.0205 (3)	
C4A	0.97819 (19)	0.49383 (12)	0.42632 (8)	0.0244 (3)	
H4A	0.908102	0.539454	0.399347	0.029*	
C5A	1.1150 (2)	0.44119 (12)	0.39235 (8)	0.0285 (3)	
H5A	1.135926	0.451866	0.343093	0.034*	
C6A	1.21719 (19)	0.37518 (12)	0.42994 (8)	0.0265 (3)	
H6A	1.308623	0.339984	0.406658	0.032*	
C7A	1.18786 (18)	0.35871 (10)	0.50358 (8)	0.0224 (3)	
C8A	1.04973 (17)	0.41166 (10)	0.53840 (8)	0.0200 (3)	
C9A	1.02091 (19)	0.39056 (11)	0.61172 (8)	0.0238 (3)	
H9A	0.931117	0.425656	0.636141	0.029*	
C10A	1.1204 (2)	0.32046 (12)	0.64783 (8)	0.0287 (3)	
H10A	1.097626	0.306807	0.696590	0.034*	
C11A	1.2563 (2)	0.26847 (12)	0.61307 (9)	0.0297 (3)	
H11A	1.324712	0.219971	0.638340	0.036*	
C12A	1.28916 (18)	0.28809 (11)	0.54313 (9)	0.0267 (3)	
H12A	1.382115	0.253617	0.520355	0.032*	
O1B	0.43141 (14)	0.71203 (8)	0.46621 (6)	0.0265 (2)	
H1B	0.525 (3)	0.6885 (16)	0.4823 (11)	0.040*	
C1B	0.23951 (19)	0.70286 (12)	0.36738 (8)	0.0281 (3)	
H1BA	0.146648	0.685713	0.400720	0.042*	
H1BB	0.218166	0.668709	0.321711	0.042*	
H1BC	0.243198	0.777652	0.360274	0.042*	
C2B	0.41076 (18)	0.66646 (11)	0.39748 (8)	0.0240 (3)	
H2B	0.408204	0.589751	0.402554	0.029*	
C3B	0.56084 (18)	0.69671 (11)	0.34933 (8)	0.0230 (3)	
C4B	0.6156 (2)	0.79748 (12)	0.34891 (9)	0.0284 (3)	
H4B	0.558720	0.846205	0.378444	0.034*	
C5B	0.7542 (2)	0.83057 (12)	0.30581 (9)	0.0307 (3)	
H5B	0.786287	0.901223	0.305330	0.037*	
C6B	0.8418 (2)	0.76161 (12)	0.26494 (9)	0.0288 (3)	
H6B	0.936383	0.784165	0.236799	0.035*	
C7B	0.79270 (18)	0.65571 (11)	0.26410 (8)	0.0234 (3)	
C8B	0.64765 (18)	0.62333 (11)	0.30537 (7)	0.0219 (3)	
C9B	0.59869 (19)	0.51755 (11)	0.30078 (8)	0.0252 (3)	
H9B	0.500839	0.494047	0.326528	0.030*	
C10B	0.6898 (2)	0.44943 (11)	0.26010 (8)	0.0268 (3)	
H10B	0.654346	0.379419	0.257880	0.032*	
C11B	0.8359 (2)	0.48176 (12)	0.22131 (8)	0.0278 (3)	
H11B	0.899890	0.433325	0.194089	0.033*	
C12B	0.88542 (19)	0.58302 (12)	0.22294 (8)	0.0268 (3)	
H12B	0.982883	0.604710	0.196209	0.032*	

### (S)-1-(Naphthalen-1-yl)ethanol (1) Atomic displacement parameters (Å^2^)

**Table:**

	*U*^11^	*U*^22^	*U*^33^	*U*^12^	*U*^13^	*U*^23^
O1A	0.0203 (5)	0.0208 (5)	0.0343 (5)	0.0024 (4)	−0.0059 (4)	0.0016 (4)
C1A	0.0173 (6)	0.0267 (7)	0.0367 (8)	−0.0001 (6)	0.0008 (6)	−0.0006 (6)
C2A	0.0166 (6)	0.0211 (6)	0.0263 (6)	0.0027 (5)	−0.0026 (5)	0.0003 (5)
C3A	0.0148 (6)	0.0192 (6)	0.0274 (6)	−0.0023 (5)	−0.0023 (5)	−0.0022 (5)
C4A	0.0200 (7)	0.0258 (7)	0.0276 (7)	−0.0031 (6)	−0.0028 (6)	−0.0004 (6)
C5A	0.0261 (8)	0.0340 (8)	0.0255 (7)	−0.0060 (6)	0.0031 (6)	−0.0040 (6)
C6A	0.0184 (7)	0.0276 (7)	0.0336 (7)	−0.0018 (6)	0.0054 (6)	−0.0083 (6)
C7A	0.0158 (6)	0.0186 (6)	0.0327 (7)	−0.0029 (5)	0.0004 (5)	−0.0060 (5)
C8A	0.0141 (6)	0.0188 (6)	0.0271 (7)	−0.0020 (5)	−0.0016 (5)	−0.0032 (5)
C9A	0.0201 (7)	0.0233 (7)	0.0280 (7)	0.0039 (5)	−0.0002 (6)	−0.0022 (6)
C10A	0.0276 (7)	0.0288 (7)	0.0297 (7)	0.0060 (6)	−0.0022 (6)	0.0014 (6)
C11A	0.0245 (8)	0.0243 (7)	0.0403 (8)	0.0063 (6)	−0.0067 (7)	0.0007 (6)
C12A	0.0169 (6)	0.0214 (6)	0.0417 (8)	0.0020 (5)	−0.0007 (6)	−0.0063 (6)
O1B	0.0205 (5)	0.0295 (5)	0.0295 (5)	0.0065 (4)	−0.0024 (4)	0.0007 (4)
C1B	0.0186 (7)	0.0312 (7)	0.0346 (8)	−0.0014 (6)	−0.0021 (6)	0.0035 (6)
C2B	0.0201 (7)	0.0243 (7)	0.0275 (7)	0.0015 (6)	0.0010 (5)	0.0015 (6)
C3B	0.0186 (6)	0.0236 (7)	0.0268 (7)	0.0005 (5)	−0.0011 (5)	0.0027 (6)
C4B	0.0236 (7)	0.0232 (7)	0.0384 (8)	0.0005 (6)	0.0009 (6)	0.0001 (6)
C5B	0.0261 (7)	0.0210 (6)	0.0450 (9)	−0.0040 (6)	0.0023 (7)	0.0018 (6)
C6B	0.0220 (7)	0.0287 (7)	0.0356 (8)	−0.0024 (6)	0.0019 (6)	0.0039 (6)
C7B	0.0177 (6)	0.0283 (7)	0.0241 (6)	0.0015 (5)	−0.0028 (5)	0.0025 (5)
C8B	0.0177 (6)	0.0252 (7)	0.0228 (6)	0.0016 (5)	−0.0032 (5)	0.0023 (5)
C9B	0.0235 (7)	0.0253 (7)	0.0270 (7)	0.0000 (6)	−0.0033 (6)	0.0017 (6)
C10B	0.0298 (8)	0.0215 (7)	0.0293 (7)	0.0015 (6)	−0.0074 (6)	−0.0016 (5)
C11B	0.0256 (7)	0.0298 (7)	0.0280 (7)	0.0062 (6)	−0.0052 (6)	−0.0037 (6)
C12B	0.0190 (7)	0.0344 (8)	0.0270 (7)	0.0023 (6)	−0.0012 (6)	−0.0003 (6)

### (S)-1-(Naphthalen-1-yl)ethanol (1) Geometric parameters (Å, º)

**Table:**

O1A—H1A	0.85 (2)	O1B—H1B	0.85 (2)
O1A—C2A	1.4315 (16)	O1B—C2B	1.4295 (18)
C1A—H1AA	0.9800	C1B—H1BA	0.9800
C1A—H1AB	0.9800	C1B—H1BB	0.9800
C1A—H1AC	0.9800	C1B—H1BC	0.9800
C1A—C2A	1.522 (2)	C1B—C2B	1.518 (2)
C2A—H2A	1.0000	C2B—H2B	1.0000
C2A—C3A	1.5230 (19)	C2B—C3B	1.525 (2)
C3A—C4A	1.374 (2)	C3B—C4B	1.375 (2)
C3A—C8A	1.4336 (19)	C3B—C8B	1.429 (2)
C4A—H4A	0.9500	C4B—H4B	0.9500
C4A—C5A	1.414 (2)	C4B—C5B	1.412 (2)
C5A—H5A	0.9500	C5B—H5B	0.9500
C5A—C6A	1.364 (2)	C5B—C6B	1.361 (2)
C6A—H6A	0.9500	C6B—H6B	0.9500
C6A—C7A	1.419 (2)	C6B—C7B	1.426 (2)
C7A—C8A	1.4306 (19)	C7B—C8B	1.429 (2)
C7A—C12A	1.417 (2)	C7B—C12B	1.416 (2)
C8A—C9A	1.423 (2)	C8B—C9B	1.427 (2)
C9A—H9A	0.9500	C9B—H9B	0.9500
C9A—C10A	1.372 (2)	C9B—C10B	1.365 (2)
C10A—H10A	0.9500	C10B—H10B	0.9500
C10A—C11A	1.411 (2)	C10B—C11B	1.411 (2)
C11A—H11A	0.9500	C11B—H11B	0.9500
C11A—C12A	1.363 (2)	C11B—C12B	1.369 (2)
C12A—H12A	0.9500	C12B—H12B	0.9500
			
C2A—O1A—H1A	109.4 (14)	C2B—O1B—H1B	105.6 (15)
H1AA—C1A—H1AB	109.5	H1BA—C1B—H1BB	109.5
H1AA—C1A—H1AC	109.5	H1BA—C1B—H1BC	109.5
H1AB—C1A—H1AC	109.5	H1BB—C1B—H1BC	109.5
C2A—C1A—H1AA	109.5	C2B—C1B—H1BA	109.5
C2A—C1A—H1AB	109.5	C2B—C1B—H1BB	109.5
C2A—C1A—H1AC	109.5	C2B—C1B—H1BC	109.5
O1A—C2A—C1A	106.67 (11)	O1B—C2B—C1B	107.81 (12)
O1A—C2A—H2A	109.3	O1B—C2B—H2B	109.1
O1A—C2A—C3A	111.46 (12)	O1B—C2B—C3B	110.13 (12)
C1A—C2A—H2A	109.3	C1B—C2B—H2B	109.1
C1A—C2A—C3A	110.88 (11)	C1B—C2B—C3B	111.45 (12)
C3A—C2A—H2A	109.3	C3B—C2B—H2B	109.1
C4A—C3A—C2A	119.87 (13)	C4B—C3B—C2B	118.94 (13)
C4A—C3A—C8A	119.50 (13)	C4B—C3B—C8B	119.00 (14)
C8A—C3A—C2A	120.49 (12)	C8B—C3B—C2B	122.06 (13)
C3A—C4A—H4A	119.3	C3B—C4B—H4B	119.1
C3A—C4A—C5A	121.43 (14)	C3B—C4B—C5B	121.84 (14)
C5A—C4A—H4A	119.3	C5B—C4B—H4B	119.1
C4A—C5A—H5A	119.8	C4B—C5B—H5B	119.9
C6A—C5A—C4A	120.34 (14)	C6B—C5B—C4B	120.24 (14)
C6A—C5A—H5A	119.8	C6B—C5B—H5B	119.9
C5A—C6A—H6A	119.8	C5B—C6B—H6B	119.8
C5A—C6A—C7A	120.46 (14)	C5B—C6B—C7B	120.42 (14)
C7A—C6A—H6A	119.8	C7B—C6B—H6B	119.8
C6A—C7A—C8A	119.59 (13)	C6B—C7B—C8B	119.18 (13)
C12A—C7A—C6A	121.35 (14)	C12B—C7B—C6B	120.82 (14)
C12A—C7A—C8A	119.02 (13)	C12B—C7B—C8B	120.01 (13)
C7A—C8A—C3A	118.67 (13)	C3B—C8B—C7B	119.24 (13)
C9A—C8A—C3A	123.38 (12)	C9B—C8B—C3B	123.41 (13)
C9A—C8A—C7A	117.92 (13)	C9B—C8B—C7B	117.34 (13)
C8A—C9A—H9A	119.4	C8B—C9B—H9B	119.4
C10A—C9A—C8A	121.22 (14)	C10B—C9B—C8B	121.26 (14)
C10A—C9A—H9A	119.4	C10B—C9B—H9B	119.4
C9A—C10A—H10A	119.7	C9B—C10B—H10B	119.6
C9A—C10A—C11A	120.51 (15)	C9B—C10B—C11B	120.81 (14)
C11A—C10A—H10A	119.7	C11B—C10B—H10B	119.6
C10A—C11A—H11A	120.1	C10B—C11B—H11B	120.0
C12A—C11A—C10A	119.77 (14)	C12B—C11B—C10B	119.91 (14)
C12A—C11A—H11A	120.1	C12B—C11B—H11B	120.0
C7A—C12A—H12A	119.2	C7B—C12B—H12B	119.7
C11A—C12A—C7A	121.55 (14)	C11B—C12B—C7B	120.62 (14)
C11A—C12A—H12A	119.2	C11B—C12B—H12B	119.7
			
O1A—C2A—C3A—C4A	−26.91 (17)	O1B—C2B—C3B—C4B	−44.17 (18)
O1A—C2A—C3A—C8A	157.32 (11)	O1B—C2B—C3B—C8B	134.99 (13)
C1A—C2A—C3A—C4A	91.76 (16)	C1B—C2B—C3B—C4B	75.44 (18)
C1A—C2A—C3A—C8A	−84.01 (16)	C1B—C2B—C3B—C8B	−105.40 (15)
C2A—C3A—C4A—C5A	−175.82 (13)	C2B—C3B—C4B—C5B	179.76 (14)
C2A—C3A—C8A—C7A	175.77 (12)	C2B—C3B—C8B—C7B	−176.97 (13)
C2A—C3A—C8A—C9A	−2.2 (2)	C2B—C3B—C8B—C9B	2.2 (2)
C3A—C4A—C5A—C6A	0.1 (2)	C3B—C4B—C5B—C6B	−2.4 (2)
C3A—C8A—C9A—C10A	176.93 (14)	C3B—C8B—C9B—C10B	−177.44 (13)
C4A—C3A—C8A—C7A	−0.01 (19)	C4B—C3B—C8B—C7B	2.2 (2)
C4A—C3A—C8A—C9A	−177.95 (13)	C4B—C3B—C8B—C9B	−178.63 (14)
C4A—C5A—C6A—C7A	−0.2 (2)	C4B—C5B—C6B—C7B	1.3 (2)
C5A—C6A—C7A—C8A	0.2 (2)	C5B—C6B—C7B—C8B	1.5 (2)
C5A—C6A—C7A—C12A	178.05 (14)	C5B—C6B—C7B—C12B	−178.64 (15)
C6A—C7A—C8A—C3A	−0.06 (19)	C6B—C7B—C8B—C3B	−3.2 (2)
C6A—C7A—C8A—C9A	177.99 (13)	C6B—C7B—C8B—C9B	177.55 (13)
C6A—C7A—C12A—C11A	−176.91 (14)	C6B—C7B—C12B—C11B	−178.84 (14)
C7A—C8A—C9A—C10A	−1.0 (2)	C7B—C8B—C9B—C10B	1.8 (2)
C8A—C3A—C4A—C5A	0.0 (2)	C8B—C3B—C4B—C5B	0.6 (2)
C8A—C7A—C12A—C11A	1.0 (2)	C8B—C7B—C12B—C11B	1.0 (2)
C8A—C9A—C10A—C11A	1.0 (2)	C8B—C9B—C10B—C11B	0.1 (2)
C9A—C10A—C11A—C12A	0.1 (2)	C9B—C10B—C11B—C12B	−1.5 (2)
C10A—C11A—C12A—C7A	−1.0 (2)	C10B—C11B—C12B—C7B	0.9 (2)
C12A—C7A—C8A—C3A	−178.00 (12)	C12B—C7B—C8B—C3B	176.92 (13)
C12A—C7A—C8A—C9A	0.05 (19)	C12B—C7B—C8B—C9B	−2.31 (19)

### (S)-1-(Naphthalen-1-yl)ethanol (1) Hydrogen-bond geometry (Å, º)

**Table:**

*D*—H···*A*	*D*—H	H···*A*	*D*···*A*	*D*—H···*A*
O1*A*—H1*A*···O1*B*^i^	0.85 (2)	1.84 (2)	2.6849 (15)	172 (2)
O1*B*—H1*B*···O1*A*	0.85 (2)	1.89 (2)	2.7119 (14)	165 (2)

Symmetry code: (i) *x*+1/2, −*y*+3/2, −*z*+1.

### (R)-1-(Naphthalen-2-yl)ethanol (2) Crystal data

**Table:**

C_12_H_12_O	*F*(000) = 736
*M**_r_* = 172.22	*D*_x_ = 1.224 Mg m^−^^3^
Monoclinic, *P*2_1_	Cu *K*α radiation, λ = 1.54178 Å
*a* = 17.3408 (5) Å	Cell parameters from 9778 reflections
*b* = 6.0327 (2) Å	θ = 2.5–78.8°
*c* = 19.1125 (5) Å	µ = 0.60 mm^−^^1^
β = 110.821 (1)°	*T* = 100 K
*V* = 1868.82 (10) Å^3^	Needle, colourless
*Z* = 8	0.56 × 0.05 × 0.04 mm

### (R)-1-(Naphthalen-2-yl)ethanol (2) Data collection

**Table:**

Bruker D8 VENTURE dual wavelength Mo/Cu diffractometer	7878 independent reflections
Radiation source: microfocus sealed X-ray tube, Incoatec Iµs	7515 reflections with *I* > 2σ(*I*)
Mirror optics monochromator	*R*_int_ = 0.051
Detector resolution: 7.41 pixels mm^-1^	θ_max_ = 79.1°, θ_min_ = 2.5°
ω and φ scans	*h* = −21→21
Absorption correction: numerical (SADABS; Krause *et al.*, 2015)	*k* = −7→7
*T*_min_ = 0.355, *T*_max_ = 0.473	*l* = −24→24
70639 measured reflections	

### (R)-1-(Naphthalen-2-yl)ethanol (2) Refinement

**Table:**

Refinement on *F*^2^	Hydrogen site location: mixed
Least-squares matrix: full	H atoms treated by a mixture of independent and constrained refinement
*R*[*F*^2^ > 2σ(*F*^2^)] = 0.035	*w* = 1/[σ^2^(*F*_o_^2^) + (0.0657*P*)^2^ + 0.2081*P*] where *P* = (*F*_o_^2^ + 2*F*_c_^2^)/3
*wR*(*F*^2^) = 0.096	(Δ/σ)_max_ < 0.001
*S* = 1.03	Δρ_max_ = 0.19 e Å^−^^3^
7878 reflections	Δρ_min_ = −0.16 e Å^−^^3^
486 parameters	Extinction correction: SHELXL-2018/3 (Sheldrick 2015b), Fc^*^=kFc[1+0.001xFc^2^λ^3^/sin(2θ)]^-1/4^
1 restraint	Extinction coefficient: 0.0019 (5)
Primary atom site location: structure-invariant direct methods	Absolute structure: Flack *x* determined using 3213 quotients [(*I*^+^)-(*I*^-^)]/[(*I*^+^)+(*I*^-^)] (Parsons *et al.*, 2013)
Secondary atom site location: difference Fourier map	Absolute structure parameter: 0.00 (15)

### (R)-1-(Naphthalen-2-yl)ethanol (2) Special details

**Table:**

Geometry. All esds (except the esd in the dihedral angle between two l.s. planes) are estimated using the full covariance matrix. The cell esds are taken into account individually in the estimation of esds in distances, angles and torsion angles; correlations between esds in cell parameters are only used when they are defined by crystal symmetry. An approximate (isotropic) treatment of cell esds is used for estimating esds involving l.s. planes.
Refinement. 1. Fixed Uiso At 1.2 times of: All C(H) groups At 1.5 times of: All C(H,H,H) groups, All O(H) groups 2.a Ternary CH refined with riding coordinates: C2A(H2A), C2B(H2B), C2C(H2C), C2D(H2D) 2.b Aromatic/amide H refined with riding coordinates: C4A(H4A), C6A(H6A), C7A(H7A), C8A(H8A), C9A(H9A), C11A(H11A), C12A(H12A), C4B(H4B), C6B(H6B), C7B(H7B), C8B(H8B), C9B(H9B), C11B(H11B), C12B(H12B), C4C(H4C), C6C(H6C), C7C(H7C), C8C(H8C), C9C(H9C), C11C(H11C), C12C(H12C), C4D(H4D), C6D(H6D), C7D(H7D), C8D(H8D), C9D(H9D), C11D(H11D), C12D(H12D) 2.c Idealised Me refined as rotating group: C1A(H1AA,H1AB,H1AC), C1B(H1BA,H1BB,H1BC), C1C(H1CA,H1CB,H1CC), C1D(H1DA,H1DB, H1DC)

### (R)-1-(Naphthalen-2-yl)ethanol (2) Fractional atomic coordinates and isotropic or equivalent isotropic displacement parameters (Å^2^)

**Table:**

	*x*	*y*	*z*	*U*_iso_*/*U*_eq_	
O1A	0.39030 (8)	−0.0225 (2)	0.72219 (7)	0.0247 (3)	
H1A	0.3483 (17)	−0.082 (5)	0.6890 (15)	0.037*	
C1A	0.53036 (12)	0.0875 (4)	0.75148 (11)	0.0309 (4)	
H1AA	0.517483	0.243671	0.756330	0.046*	
H1AB	0.578269	0.077708	0.735814	0.046*	
H1AC	0.542826	0.012981	0.799818	0.046*	
C2A	0.45672 (10)	−0.0237 (3)	0.69335 (10)	0.0236 (3)	
H2A	0.471455	−0.180918	0.687188	0.028*	
C3A	0.43001 (10)	0.0900 (3)	0.61778 (10)	0.0210 (3)	
C4A	0.43768 (10)	−0.0146 (3)	0.55675 (10)	0.0204 (3)	
H4A	0.462998	−0.156484	0.562797	0.024*	
C5A	0.40842 (10)	0.0855 (3)	0.48470 (10)	0.0195 (3)	
C6A	0.41427 (10)	−0.0224 (3)	0.42066 (10)	0.0230 (3)	
H6A	0.440380	−0.162958	0.425740	0.028*	
C7A	0.38245 (11)	0.0755 (3)	0.35172 (10)	0.0257 (4)	
H7A	0.387114	0.002842	0.309310	0.031*	
C8A	0.34270 (11)	0.2838 (3)	0.34297 (10)	0.0259 (4)	
H8A	0.319563	0.348068	0.294596	0.031*	
C9A	0.33738 (11)	0.3933 (3)	0.40387 (10)	0.0238 (4)	
H9A	0.311400	0.534270	0.397548	0.029*	
C10A	0.37034 (10)	0.2978 (3)	0.47634 (10)	0.0203 (3)	
C11A	0.36459 (10)	0.4047 (3)	0.54063 (10)	0.0224 (3)	
H11A	0.340340	0.547741	0.535878	0.027*	
C12A	0.39349 (11)	0.3042 (3)	0.60911 (10)	0.0226 (4)	
H12A	0.389163	0.378250	0.651431	0.027*	
O1B	0.31560 (9)	0.3220 (3)	0.76906 (7)	0.0328 (3)	
H1B	0.3481 (19)	0.231 (6)	0.7609 (16)	0.049*	
C1B	0.43974 (13)	0.5252 (4)	0.84375 (12)	0.0332 (4)	
H1BA	0.474744	0.393023	0.852326	0.050*	
H1BB	0.461417	0.625807	0.886558	0.050*	
H1BC	0.439438	0.600540	0.798229	0.050*	
C2B	0.35249 (11)	0.4574 (3)	0.83469 (10)	0.0273 (4)	
H2B	0.318870	0.595860	0.827838	0.033*	
C3B	0.34888 (11)	0.3404 (3)	0.90343 (10)	0.0243 (4)	
C4B	0.31125 (11)	0.4384 (3)	0.94742 (10)	0.0250 (4)	
H4B	0.286739	0.580409	0.933981	0.030*	
C5B	0.30800 (11)	0.3326 (3)	1.01283 (10)	0.0252 (4)	
C6B	0.26646 (12)	0.4295 (4)	1.05733 (11)	0.0323 (4)	
H6B	0.242253	0.572232	1.044957	0.039*	
C7B	0.26112 (13)	0.3187 (4)	1.11786 (12)	0.0388 (5)	
H7B	0.232991	0.384551	1.147119	0.047*	
C8B	0.29710 (13)	0.1070 (5)	1.13716 (11)	0.0383 (5)	
H8B	0.292768	0.031102	1.179160	0.046*	
C9B	0.33829 (12)	0.0101 (4)	1.09572 (11)	0.0329 (4)	
H9B	0.362970	−0.131504	1.109698	0.039*	
C10B	0.34433 (11)	0.1194 (3)	1.03221 (10)	0.0269 (4)	
C11B	0.38353 (11)	0.0206 (3)	0.98591 (11)	0.0277 (4)	
H11B	0.408560	−0.121073	0.998529	0.033*	
C12B	0.38551 (12)	0.1275 (3)	0.92345 (11)	0.0269 (4)	
H12B	0.411677	0.058796	0.893052	0.032*	
O1C	0.18245 (7)	0.4837 (2)	0.66222 (6)	0.0207 (2)	
H1C	0.2289 (16)	0.430 (4)	0.6976 (14)	0.031*	
C1C	0.03587 (10)	0.4844 (3)	0.62168 (10)	0.0245 (4)	
H1CA	0.033933	0.640010	0.606415	0.037*	
H1CB	−0.011522	0.452094	0.636555	0.037*	
H1CC	0.034009	0.388632	0.579692	0.037*	
C2C	0.11532 (10)	0.4417 (3)	0.68743 (9)	0.0196 (3)	
H2C	0.116868	0.282329	0.702238	0.024*	
C3C	0.12073 (10)	0.5853 (3)	0.75442 (9)	0.0191 (3)	
C4C	0.08734 (10)	0.5134 (3)	0.80584 (9)	0.0199 (3)	
H4C	0.061745	0.371894	0.799662	0.024*	
C5C	0.09049 (10)	0.6471 (3)	0.86803 (9)	0.0191 (3)	
C6C	0.05706 (11)	0.5760 (3)	0.92223 (10)	0.0223 (3)	
H6C	0.030332	0.436205	0.916673	0.027*	
C7C	0.06309 (12)	0.7078 (3)	0.98266 (10)	0.0251 (4)	
H7C	0.040990	0.657811	1.018778	0.030*	
C8C	0.10188 (11)	0.9169 (3)	0.99138 (10)	0.0243 (4)	
H8C	0.105756	1.006899	1.033293	0.029*	
C9C	0.13396 (10)	0.9906 (3)	0.93964 (10)	0.0229 (3)	
H9C	0.159455	1.132250	0.945779	0.027*	
C10C	0.12965 (10)	0.8585 (3)	0.87711 (9)	0.0197 (3)	
C11C	0.16374 (10)	0.9291 (3)	0.82350 (10)	0.0215 (3)	
H11C	0.189978	1.069636	0.828990	0.026*	
C12C	0.15923 (11)	0.7969 (3)	0.76385 (10)	0.0213 (3)	
H12C	0.182136	0.847321	0.728339	0.026*	
O1D	0.26221 (8)	0.7902 (2)	0.60343 (7)	0.0219 (3)	
H1D	0.2286 (16)	0.710 (5)	0.6173 (14)	0.033*	
C1D	0.14051 (12)	1.0235 (3)	0.55515 (10)	0.0246 (4)	
H1DA	0.103942	0.904295	0.559262	0.037*	
H1DB	0.110261	1.121687	0.513614	0.037*	
H1DC	0.159932	1.108472	0.601878	0.037*	
C2D	0.21417 (10)	0.9238 (3)	0.54066 (9)	0.0199 (3)	
H2D	0.249813	1.048575	0.535640	0.024*	
C3D	0.18596 (10)	0.7947 (3)	0.46782 (9)	0.0183 (3)	
C4D	0.19249 (10)	0.8838 (3)	0.40404 (9)	0.0185 (3)	
H4D	0.219660	1.022107	0.406867	0.022*	
C5D	0.15950 (10)	0.7738 (3)	0.33394 (9)	0.0189 (3)	
C6D	0.16599 (11)	0.8622 (3)	0.26731 (10)	0.0235 (4)	
H6D	0.193010	1.000065	0.268862	0.028*	
C7D	0.13354 (12)	0.7499 (3)	0.20069 (10)	0.0275 (4)	
H7D	0.138461	0.810695	0.156610	0.033*	
C8D	0.09286 (12)	0.5448 (3)	0.19713 (10)	0.0276 (4)	
H8D	0.070436	0.468750	0.150737	0.033*	
C9D	0.08573 (11)	0.4556 (3)	0.26030 (10)	0.0248 (4)	
H9D	0.057911	0.318300	0.257387	0.030*	
C10D	0.11944 (10)	0.5659 (3)	0.33018 (9)	0.0201 (3)	
C11D	0.11518 (11)	0.4746 (3)	0.39733 (10)	0.0231 (3)	
H11D	0.089541	0.334646	0.395923	0.028*	
C12D	0.14744 (11)	0.5853 (3)	0.46390 (10)	0.0222 (3)	
H12D	0.144006	0.521035	0.508076	0.027*	

### (R)-1-(Naphthalen-2-yl)ethanol (2) Atomic displacement parameters (Å^2^)

**Table:**

	*U*^11^	*U*^22^	*U*^33^	*U*^12^	*U*^13^	*U*^23^
O1A	0.0227 (6)	0.0283 (7)	0.0217 (6)	−0.0025 (5)	0.0064 (5)	−0.0004 (5)
C1A	0.0237 (9)	0.0373 (10)	0.0277 (9)	0.0007 (8)	0.0043 (7)	−0.0029 (8)
C2A	0.0223 (8)	0.0245 (8)	0.0231 (8)	0.0017 (7)	0.0070 (7)	0.0000 (7)
C3A	0.0152 (7)	0.0219 (8)	0.0242 (8)	−0.0020 (6)	0.0050 (6)	0.0001 (7)
C4A	0.0162 (7)	0.0183 (8)	0.0254 (8)	0.0002 (6)	0.0060 (6)	0.0005 (6)
C5A	0.0133 (7)	0.0192 (8)	0.0249 (8)	−0.0030 (6)	0.0053 (6)	−0.0011 (6)
C6A	0.0184 (7)	0.0222 (8)	0.0274 (8)	−0.0008 (6)	0.0071 (6)	−0.0012 (7)
C7A	0.0217 (8)	0.0305 (9)	0.0242 (9)	−0.0035 (7)	0.0074 (7)	−0.0038 (7)
C8A	0.0209 (8)	0.0297 (10)	0.0243 (8)	−0.0036 (7)	0.0046 (7)	0.0056 (7)
C9A	0.0175 (8)	0.0214 (8)	0.0303 (9)	−0.0008 (6)	0.0058 (7)	0.0040 (7)
C10A	0.0142 (7)	0.0198 (8)	0.0267 (8)	−0.0032 (6)	0.0068 (6)	0.0010 (7)
C11A	0.0183 (8)	0.0175 (8)	0.0318 (9)	0.0002 (6)	0.0093 (7)	−0.0007 (7)
C12A	0.0208 (8)	0.0213 (8)	0.0273 (9)	−0.0020 (6)	0.0103 (7)	−0.0046 (7)
O1B	0.0269 (7)	0.0413 (8)	0.0232 (6)	0.0112 (6)	0.0001 (5)	−0.0036 (6)
C1B	0.0309 (10)	0.0367 (11)	0.0307 (10)	0.0014 (8)	0.0093 (8)	0.0062 (8)
C2B	0.0261 (9)	0.0276 (9)	0.0236 (8)	0.0069 (7)	0.0032 (7)	0.0014 (7)
C3B	0.0190 (8)	0.0255 (9)	0.0238 (8)	0.0006 (7)	0.0019 (7)	−0.0010 (7)
C4B	0.0198 (8)	0.0209 (8)	0.0293 (9)	0.0006 (6)	0.0024 (7)	−0.0034 (7)
C5B	0.0171 (8)	0.0294 (9)	0.0255 (9)	−0.0025 (7)	0.0032 (7)	−0.0058 (7)
C6B	0.0231 (9)	0.0389 (11)	0.0318 (10)	−0.0011 (8)	0.0060 (7)	−0.0103 (8)
C7B	0.0256 (10)	0.0601 (14)	0.0289 (10)	−0.0060 (10)	0.0076 (8)	−0.0109 (10)
C8B	0.0276 (10)	0.0597 (15)	0.0238 (9)	−0.0109 (10)	0.0046 (8)	0.0019 (9)
C9B	0.0240 (9)	0.0386 (11)	0.0293 (9)	−0.0049 (8)	0.0014 (7)	0.0050 (8)
C10B	0.0189 (8)	0.0312 (10)	0.0254 (9)	−0.0036 (7)	0.0014 (7)	−0.0015 (7)
C11B	0.0233 (9)	0.0238 (9)	0.0303 (9)	0.0019 (7)	0.0024 (7)	−0.0004 (7)
C12B	0.0240 (9)	0.0253 (9)	0.0280 (9)	0.0045 (7)	0.0052 (7)	−0.0035 (7)
O1C	0.0177 (5)	0.0266 (6)	0.0184 (5)	0.0022 (5)	0.0071 (4)	0.0013 (5)
C1C	0.0196 (8)	0.0290 (9)	0.0243 (8)	−0.0003 (7)	0.0071 (7)	−0.0035 (7)
C2C	0.0207 (8)	0.0190 (8)	0.0207 (8)	0.0003 (6)	0.0092 (6)	0.0001 (6)
C3C	0.0173 (7)	0.0194 (8)	0.0197 (8)	0.0018 (6)	0.0057 (6)	0.0007 (6)
C4C	0.0188 (8)	0.0177 (8)	0.0223 (8)	0.0007 (6)	0.0062 (6)	0.0010 (6)
C5C	0.0165 (8)	0.0197 (8)	0.0193 (8)	0.0018 (6)	0.0043 (6)	0.0014 (6)
C6C	0.0206 (8)	0.0234 (8)	0.0229 (8)	−0.0002 (6)	0.0078 (7)	0.0006 (7)
C7C	0.0238 (9)	0.0315 (9)	0.0206 (8)	0.0022 (7)	0.0088 (7)	0.0012 (7)
C8C	0.0215 (8)	0.0296 (9)	0.0195 (8)	0.0024 (7)	0.0046 (6)	−0.0049 (7)
C9C	0.0187 (8)	0.0219 (8)	0.0249 (8)	0.0010 (7)	0.0037 (6)	−0.0032 (7)
C10C	0.0163 (7)	0.0202 (8)	0.0208 (8)	0.0023 (6)	0.0043 (6)	0.0009 (6)
C11C	0.0203 (8)	0.0177 (8)	0.0256 (8)	−0.0008 (6)	0.0072 (7)	0.0001 (6)
C12C	0.0204 (8)	0.0217 (8)	0.0230 (8)	0.0004 (6)	0.0090 (7)	0.0026 (6)
O1D	0.0209 (6)	0.0242 (6)	0.0191 (6)	−0.0022 (5)	0.0050 (5)	0.0009 (5)
C1D	0.0294 (9)	0.0223 (9)	0.0226 (8)	0.0031 (7)	0.0098 (7)	−0.0020 (7)
C2D	0.0223 (8)	0.0188 (8)	0.0177 (7)	−0.0029 (6)	0.0060 (6)	−0.0012 (6)
C3D	0.0159 (7)	0.0191 (8)	0.0193 (7)	0.0009 (6)	0.0056 (6)	−0.0019 (6)
C4D	0.0170 (7)	0.0167 (7)	0.0213 (8)	−0.0011 (6)	0.0061 (6)	−0.0002 (6)
C5D	0.0159 (8)	0.0210 (8)	0.0198 (8)	0.0016 (6)	0.0062 (6)	0.0007 (6)
C6D	0.0222 (8)	0.0266 (9)	0.0219 (8)	−0.0020 (7)	0.0083 (7)	0.0021 (7)
C7D	0.0282 (10)	0.0348 (10)	0.0190 (8)	−0.0011 (8)	0.0078 (7)	0.0018 (7)
C8D	0.0265 (9)	0.0342 (10)	0.0190 (8)	−0.0013 (7)	0.0044 (7)	−0.0063 (7)
C9D	0.0239 (8)	0.0242 (9)	0.0236 (8)	−0.0030 (7)	0.0050 (7)	−0.0033 (7)
C10D	0.0184 (8)	0.0213 (8)	0.0196 (8)	0.0001 (6)	0.0053 (6)	−0.0011 (6)
C11D	0.0251 (8)	0.0191 (8)	0.0245 (8)	−0.0046 (7)	0.0080 (7)	−0.0013 (7)
C12D	0.0251 (8)	0.0212 (8)	0.0209 (8)	−0.0021 (7)	0.0088 (7)	0.0011 (6)

### (R)-1-(Naphthalen-2-yl)ethanol (2) Geometric parameters (Å, º)

**Table:**

O1A—H1A	0.86 (3)	O1C—H1C	0.91 (3)
O1A—C2A	1.443 (2)	O1C—C2C	1.4318 (19)
C1A—H1AA	0.9800	C1C—H1CA	0.9800
C1A—H1AB	0.9800	C1C—H1CB	0.9800
C1A—H1AC	0.9800	C1C—H1CC	0.9800
C1A—C2A	1.519 (3)	C1C—C2C	1.521 (2)
C2A—H2A	1.0000	C2C—H2C	1.0000
C2A—C3A	1.515 (2)	C2C—C3C	1.521 (2)
C3A—C4A	1.374 (2)	C3C—C4C	1.376 (2)
C3A—C12A	1.423 (2)	C3C—C12C	1.422 (2)
C4A—H4A	0.9500	C4C—H4C	0.9500
C4A—C5A	1.422 (2)	C4C—C5C	1.422 (2)
C5A—C6A	1.421 (2)	C5C—C6C	1.421 (2)
C5A—C10A	1.424 (2)	C5C—C10C	1.426 (2)
C6A—H6A	0.9500	C6C—H6C	0.9500
C6A—C7A	1.368 (3)	C6C—C7C	1.375 (3)
C7A—H7A	0.9500	C7C—H7C	0.9500
C7A—C8A	1.414 (3)	C7C—C8C	1.411 (3)
C8A—H8A	0.9500	C8C—H8C	0.9500
C8A—C9A	1.370 (3)	C8C—C9C	1.370 (3)
C9A—H9A	0.9500	C9C—H9C	0.9500
C9A—C10A	1.419 (2)	C9C—C10C	1.416 (2)
C10A—C11A	1.422 (2)	C10C—C11C	1.418 (2)
C11A—H11A	0.9500	C11C—H11C	0.9500
C11A—C12A	1.366 (3)	C11C—C12C	1.371 (2)
C12A—H12A	0.9500	C12C—H12C	0.9500
O1B—H1B	0.84 (3)	O1D—H1D	0.87 (3)
O1B—C2B	1.442 (2)	O1D—C2D	1.440 (2)
C1B—H1BA	0.9800	C1D—H1DA	0.9800
C1B—H1BB	0.9800	C1D—H1DB	0.9800
C1B—H1BC	0.9800	C1D—H1DC	0.9800
C1B—C2B	1.517 (3)	C1D—C2D	1.524 (2)
C2B—H2B	1.0000	C2D—H2D	1.0000
C2B—C3B	1.512 (3)	C2D—C3D	1.517 (2)
C3B—C4B	1.369 (3)	C3D—C4D	1.373 (2)
C3B—C12B	1.424 (3)	C3D—C12D	1.419 (2)
C4B—H4B	0.9500	C4D—H4D	0.9500
C4B—C5B	1.423 (3)	C4D—C5D	1.421 (2)
C5B—C6B	1.421 (3)	C5D—C6D	1.421 (2)
C5B—C10B	1.422 (3)	C5D—C10D	1.423 (2)
C6B—H6B	0.9500	C6D—H6D	0.9500
C6B—C7B	1.368 (3)	C6D—C7D	1.374 (3)
C7B—H7B	0.9500	C7D—H7D	0.9500
C7B—C8B	1.412 (4)	C7D—C8D	1.414 (3)
C8B—H8B	0.9500	C8D—H8D	0.9500
C8B—C9B	1.372 (3)	C8D—C9D	1.367 (3)
C9B—H9B	0.9500	C9D—H9D	0.9500
C9B—C10B	1.418 (3)	C9D—C10D	1.419 (2)
C10B—C11B	1.424 (3)	C10D—C11D	1.422 (2)
C11B—H11B	0.9500	C11D—H11D	0.9500
C11B—C12B	1.368 (3)	C11D—C12D	1.368 (2)
C12B—H12B	0.9500	C12D—H12D	0.9500
			
C2A—O1A—H1A	107.4 (17)	C2C—O1C—H1C	107.4 (15)
H1AA—C1A—H1AB	109.5	H1CA—C1C—H1CB	109.5
H1AA—C1A—H1AC	109.5	H1CA—C1C—H1CC	109.5
H1AB—C1A—H1AC	109.5	H1CB—C1C—H1CC	109.5
C2A—C1A—H1AA	109.5	C2C—C1C—H1CA	109.5
C2A—C1A—H1AB	109.5	C2C—C1C—H1CB	109.5
C2A—C1A—H1AC	109.5	C2C—C1C—H1CC	109.5
O1A—C2A—C1A	107.21 (14)	O1C—C2C—C1C	107.34 (13)
O1A—C2A—H2A	108.8	O1C—C2C—H2C	108.8
O1A—C2A—C3A	110.30 (14)	O1C—C2C—C3C	111.61 (13)
C1A—C2A—H2A	108.8	C1C—C2C—H2C	108.8
C3A—C2A—C1A	112.95 (16)	C1C—C2C—C3C	111.47 (14)
C3A—C2A—H2A	108.8	C3C—C2C—H2C	108.8
C4A—C3A—C2A	120.81 (16)	C4C—C3C—C2C	120.39 (15)
C4A—C3A—C12A	119.47 (16)	C4C—C3C—C12C	119.36 (15)
C12A—C3A—C2A	119.67 (16)	C12C—C3C—C2C	120.25 (15)
C3A—C4A—H4A	119.4	C3C—C4C—H4C	119.4
C3A—C4A—C5A	121.21 (16)	C3C—C4C—C5C	121.16 (16)
C5A—C4A—H4A	119.4	C5C—C4C—H4C	119.4
C4A—C5A—C10A	118.85 (16)	C4C—C5C—C10C	118.97 (15)
C6A—C5A—C4A	121.96 (16)	C6C—C5C—C4C	122.26 (16)
C6A—C5A—C10A	119.18 (16)	C6C—C5C—C10C	118.77 (15)
C5A—C6A—H6A	119.9	C5C—C6C—H6C	119.7
C7A—C6A—C5A	120.25 (17)	C7C—C6C—C5C	120.57 (17)
C7A—C6A—H6A	119.9	C7C—C6C—H6C	119.7
C6A—C7A—H7A	119.7	C6C—C7C—H7C	119.8
C6A—C7A—C8A	120.67 (17)	C6C—C7C—C8C	120.45 (17)
C8A—C7A—H7A	119.7	C8C—C7C—H7C	119.8
C7A—C8A—H8A	119.8	C7C—C8C—H8C	119.9
C9A—C8A—C7A	120.34 (17)	C9C—C8C—C7C	120.24 (17)
C9A—C8A—H8A	119.8	C9C—C8C—H8C	119.9
C8A—C9A—H9A	119.7	C8C—C9C—H9C	119.6
C8A—C9A—C10A	120.55 (17)	C8C—C9C—C10C	120.90 (17)
C10A—C9A—H9A	119.7	C10C—C9C—H9C	119.6
C9A—C10A—C5A	118.98 (16)	C9C—C10C—C5C	119.07 (15)
C9A—C10A—C11A	122.11 (16)	C9C—C10C—C11C	122.00 (16)
C11A—C10A—C5A	118.89 (16)	C11C—C10C—C5C	118.92 (15)
C10A—C11A—H11A	119.6	C10C—C11C—H11C	119.6
C12A—C11A—C10A	120.81 (16)	C12C—C11C—C10C	120.72 (16)
C12A—C11A—H11A	119.6	C12C—C11C—H11C	119.6
C3A—C12A—H12A	119.6	C3C—C12C—H12C	119.6
C11A—C12A—C3A	120.75 (16)	C11C—C12C—C3C	120.88 (16)
C11A—C12A—H12A	119.6	C11C—C12C—H12C	119.6
C2B—O1B—H1B	114 (2)	C2D—O1D—H1D	108.3 (17)
H1BA—C1B—H1BB	109.5	H1DA—C1D—H1DB	109.5
H1BA—C1B—H1BC	109.5	H1DA—C1D—H1DC	109.5
H1BB—C1B—H1BC	109.5	H1DB—C1D—H1DC	109.5
C2B—C1B—H1BA	109.5	C2D—C1D—H1DA	109.5
C2B—C1B—H1BB	109.5	C2D—C1D—H1DB	109.5
C2B—C1B—H1BC	109.5	C2D—C1D—H1DC	109.5
O1B—C2B—C1B	111.60 (16)	O1D—C2D—C1D	110.11 (13)
O1B—C2B—H2B	107.5	O1D—C2D—H2D	107.9
O1B—C2B—C3B	110.39 (16)	O1D—C2D—C3D	112.32 (13)
C1B—C2B—H2B	107.5	C1D—C2D—H2D	107.9
C3B—C2B—C1B	111.99 (15)	C3D—C2D—C1D	110.59 (14)
C3B—C2B—H2B	107.5	C3D—C2D—H2D	107.9
C4B—C3B—C2B	120.52 (17)	C4D—C3D—C2D	120.81 (15)
C4B—C3B—C12B	119.31 (17)	C4D—C3D—C12D	119.19 (15)
C12B—C3B—C2B	120.16 (17)	C12D—C3D—C2D	119.87 (15)
C3B—C4B—H4B	119.3	C3D—C4D—H4D	119.3
C3B—C4B—C5B	121.49 (17)	C3D—C4D—C5D	121.43 (15)
C5B—C4B—H4B	119.3	C5D—C4D—H4D	119.3
C6B—C5B—C4B	121.86 (18)	C4D—C5D—C6D	122.42 (16)
C6B—C5B—C10B	119.23 (18)	C4D—C5D—C10D	119.02 (15)
C10B—C5B—C4B	118.85 (17)	C6D—C5D—C10D	118.55 (15)
C5B—C6B—H6B	119.8	C5D—C6D—H6D	119.7
C7B—C6B—C5B	120.4 (2)	C7D—C6D—C5D	120.58 (17)
C7B—C6B—H6B	119.8	C7D—C6D—H6D	119.7
C6B—C7B—H7B	119.8	C6D—C7D—H7D	119.7
C6B—C7B—C8B	120.5 (2)	C6D—C7D—C8D	120.60 (17)
C8B—C7B—H7B	119.8	C8D—C7D—H7D	119.7
C7B—C8B—H8B	119.8	C7D—C8D—H8D	119.9
C9B—C8B—C7B	120.5 (2)	C9D—C8D—C7D	120.14 (16)
C9B—C8B—H8B	119.8	C9D—C8D—H8D	119.9
C8B—C9B—H9B	119.7	C8D—C9D—H9D	119.7
C8B—C9B—C10B	120.5 (2)	C8D—C9D—C10D	120.69 (17)
C10B—C9B—H9B	119.7	C10D—C9D—H9D	119.7
C5B—C10B—C11B	118.81 (17)	C9D—C10D—C5D	119.43 (16)
C9B—C10B—C5B	118.88 (18)	C9D—C10D—C11D	122.10 (16)
C9B—C10B—C11B	122.29 (19)	C11D—C10D—C5D	118.47 (15)
C10B—C11B—H11B	119.6	C10D—C11D—H11D	119.5
C12B—C11B—C10B	120.70 (18)	C12D—C11D—C10D	120.98 (16)
C12B—C11B—H11B	119.6	C12D—C11D—H11D	119.5
C3B—C12B—H12B	119.6	C3D—C12D—H12D	119.6
C11B—C12B—C3B	120.84 (17)	C11D—C12D—C3D	120.87 (16)
C11B—C12B—H12B	119.6	C11D—C12D—H12D	119.6
			
O1A—C2A—C3A—C4A	−126.61 (17)	O1C—C2C—C3C—C4C	−152.07 (15)
O1A—C2A—C3A—C12A	50.9 (2)	O1C—C2C—C3C—C12C	28.9 (2)
C1A—C2A—C3A—C4A	113.44 (19)	C1C—C2C—C3C—C4C	87.92 (19)
C1A—C2A—C3A—C12A	−69.1 (2)	C1C—C2C—C3C—C12C	−91.07 (19)
C2A—C3A—C4A—C5A	176.32 (14)	C2C—C3C—C4C—C5C	−178.83 (14)
C2A—C3A—C12A—C11A	−176.19 (15)	C2C—C3C—C12C—C11C	179.27 (15)
C3A—C4A—C5A—C6A	−178.65 (15)	C3C—C4C—C5C—C6C	−179.49 (16)
C3A—C4A—C5A—C10A	−0.2 (2)	C3C—C4C—C5C—C10C	−0.5 (2)
C4A—C3A—C12A—C11A	1.3 (3)	C4C—C3C—C12C—C11C	0.3 (2)
C4A—C5A—C6A—C7A	177.39 (16)	C4C—C5C—C6C—C7C	178.39 (16)
C4A—C5A—C10A—C9A	−176.88 (15)	C4C—C5C—C10C—C9C	−179.05 (15)
C4A—C5A—C10A—C11A	1.4 (2)	C4C—C5C—C10C—C11C	0.4 (2)
C5A—C6A—C7A—C8A	−0.6 (3)	C5C—C6C—C7C—C8C	0.6 (3)
C5A—C10A—C11A—C12A	−1.3 (2)	C5C—C10C—C11C—C12C	0.0 (2)
C6A—C5A—C10A—C9A	1.6 (2)	C6C—C5C—C10C—C9C	0.0 (2)
C6A—C5A—C10A—C11A	179.92 (15)	C6C—C5C—C10C—C11C	179.43 (15)
C6A—C7A—C8A—C9A	1.7 (3)	C6C—C7C—C8C—C9C	0.0 (3)
C7A—C8A—C9A—C10A	−1.1 (3)	C7C—C8C—C9C—C10C	−0.6 (3)
C8A—C9A—C10A—C5A	−0.5 (2)	C8C—C9C—C10C—C5C	0.7 (2)
C8A—C9A—C10A—C11A	−178.80 (16)	C8C—C9C—C10C—C11C	−178.81 (16)
C9A—C10A—C11A—C12A	176.95 (16)	C9C—C10C—C11C—C12C	179.46 (16)
C10A—C5A—C6A—C7A	−1.0 (2)	C10C—C5C—C6C—C7C	−0.6 (3)
C10A—C11A—C12A—C3A	−0.1 (3)	C10C—C11C—C12C—C3C	−0.3 (3)
C12A—C3A—C4A—C5A	−1.2 (3)	C12C—C3C—C4C—C5C	0.2 (2)
O1B—C2B—C3B—C4B	−123.14 (18)	O1D—C2D—C3D—C4D	−134.67 (16)
O1B—C2B—C3B—C12B	57.6 (2)	O1D—C2D—C3D—C12D	49.4 (2)
C1B—C2B—C3B—C4B	111.9 (2)	C1D—C2D—C3D—C4D	101.89 (18)
C1B—C2B—C3B—C12B	−67.4 (2)	C1D—C2D—C3D—C12D	−74.01 (19)
C2B—C3B—C4B—C5B	−179.22 (15)	C2D—C3D—C4D—C5D	−174.15 (15)
C2B—C3B—C12B—C11B	179.29 (17)	C2D—C3D—C12D—C11D	174.36 (16)
C3B—C4B—C5B—C6B	−177.67 (17)	C3D—C4D—C5D—C6D	−179.58 (16)
C3B—C4B—C5B—C10B	−0.5 (3)	C3D—C4D—C5D—C10D	−0.5 (2)
C4B—C3B—C12B—C11B	0.0 (3)	C4D—C3D—C12D—C11D	−1.6 (3)
C4B—C5B—C6B—C7B	176.88 (17)	C4D—C5D—C6D—C7D	179.71 (17)
C4B—C5B—C10B—C9B	−177.55 (16)	C4D—C5D—C10D—C9D	179.51 (16)
C4B—C5B—C10B—C11B	0.7 (2)	C4D—C5D—C10D—C11D	−1.0 (2)
C5B—C6B—C7B—C8B	0.3 (3)	C5D—C6D—C7D—C8D	0.2 (3)
C5B—C10B—C11B—C12B	−0.7 (3)	C5D—C10D—C11D—C12D	1.2 (3)
C6B—C5B—C10B—C9B	−0.3 (3)	C6D—C5D—C10D—C9D	−1.4 (2)
C6B—C5B—C10B—C11B	178.02 (17)	C6D—C5D—C10D—C11D	178.15 (16)
C6B—C7B—C8B—C9B	0.3 (3)	C6D—C7D—C8D—C9D	−0.2 (3)
C7B—C8B—C9B—C10B	−0.9 (3)	C7D—C8D—C9D—C10D	−0.5 (3)
C8B—C9B—C10B—C5B	0.9 (3)	C8D—C9D—C10D—C5D	1.3 (3)
C8B—C9B—C10B—C11B	−177.32 (19)	C8D—C9D—C10D—C11D	−178.16 (17)
C9B—C10B—C11B—C12B	177.54 (17)	C9D—C10D—C11D—C12D	−179.34 (16)
C10B—C5B—C6B—C7B	−0.3 (3)	C10D—C5D—C6D—C7D	0.6 (3)
C10B—C11B—C12B—C3B	0.3 (3)	C10D—C11D—C12D—C3D	0.1 (3)
C12B—C3B—C4B—C5B	0.1 (3)	C12D—C3D—C4D—C5D	1.8 (2)

### (R)-1-(Naphthalen-2-yl)ethanol (2) Hydrogen-bond geometry (Å, º)

**Table:**

*D*—H···*A*	*D*—H	H···*A*	*D*···*A*	*D*—H···*A*
O1*A*—H1*A*···O1*D*^i^	0.86 (3)	1.94 (3)	2.7890 (18)	171 (2)
O1*B*—H1*B*···O1*A*	0.84 (3)	1.95 (3)	2.761 (2)	162 (3)
O1*C*—H1*C*···O1*B*	0.91 (3)	1.76 (3)	2.6648 (18)	177 (2)
O1*D*—H1*D*···O1*C*	0.87 (3)	1.93 (3)	2.7747 (18)	163 (2)

Symmetry code: (i) *x*, *y*−1, *z*.
